# Malaria knowledge and long-lasting insecticidal net use in rural communities of central Côte d'Ivoire

**DOI:** 10.1186/1475-2875-10-288

**Published:** 2011-10-04

**Authors:** Allassane F Ouattara, Giovanna Raso, Constant VA Edi, Jürg Utzinger, Marcel Tanner, Mamadou Dagnogo, Benjamin G Koudou

**Affiliations:** 1 Département Environnement et Santé, Centre Suisse de Recherches Scientifiques en Côte d'Ivoire, 01 BP 1303, Abidjan 01, Côte d'Ivoire; 2 Laboratoire de Cytologie et de Biologie Animale, UFR Sciences de la Nature, Université d'Abobo-Adjamé, Abidjan, Côte d'Ivoire; 3 Department of Epidemiology and Public Health, Swiss Tropical and Public Health Institute, Basel, Switzerland; 4 University of Basel, Basel, Switzerland; 5 Vector Group, Liverpool School of Tropical Medicine, Liverpool, UK

## Abstract

**Background:**

To improve effectiveness of malaria control interventions, it is essential to deepen the knowledge of contextual factors that govern people's practice for preventive and curative measures. The aim of this study was to determine factors that influence the use of long-lasting insecticidal nets (LLINs) in three rural communities of Côte d'Ivoire, two of which benefited from recent interventions.

**Methods:**

The study was carried out in 957 households in three villages (Bozi, N'Dakonankro and Yoho) located in central Côte d'Ivoire. Indicators of socioeconomic position (SEP), malaria knowledge and practice, placing special emphasis on LLINs, were investigated during a cross-sectional questionnaire survey. Principal component analysis was used to calculate the SEP of households by means of a list of household assets ownership. The concentration index was used to assess the direction of the association between SEP and a given variable. To compare groups or means, Fisher's exact test, χ^2 ^and Kruskal-Wallis test were used, as appropriate.

**Results:**

Significant differences were found between SEP and reported malaria symptoms, such as fever or hot body, convulsion, anaemia and jaundice (yellow eyes). Individuals from the least poor group cited more often the use of bed nets and insecticide-treated nets (ITNs) compared to poorer groups. The mean number of individuals reporting the use of bed nets and LLINs was different between groups with different educational level. Moreover, the mean number of LLINs in a household was influenced by the presence of children below five years of age.

**Conclusion:**

The study not only confirmed that education and SEP play important roles in the prevention and control of malaria and promotion of health in general, but pointed at the basic essential knowledge and the key behavioural elements that should guide education and learning processes among the poorer segments of the population. In turn, such knowledge may change behaviour and lead to an increased utilization of LLINs.

## Background

Malaria is a vector-borne disease that is currently endemic in 109 countries [1]. In highly malaria endemic areas, such as sub-Saharan Africa, preschool-aged children and pregnant women are at highest risk because their anti-malarial immunity level is low [2]. Furthermore, malaria delays social and economic development, affecting especially the poorest segments of the population [3]. Indeed, more than half of global malaria deaths are concentrated among the world's poorest 20%. In terms of global burden estimates, 58% of the disability-adjusted life years (DALYs) due to malaria are concentrated in the poorest quintile of the global population [4]. A significant reduction of mortality, morbidity and economic losses could be achieved if control interventions with a proven track record could be implemented in areas of highest need, including insecticide-treated nets (ITNs), long-lasting insecticidal nets (LLINs), prompt diagnosis and effective treatment using artemisinin-based combination therapy (ACT) [1,5,6].

A high coverage of bed nets, particularly those treated with an insecticide (ITNs and LLINs), results in a decrease in malaria mortality and morbidity and reduces transmission [2], as shown in studies from rural Côte d'Ivoire [7] and other parts of Africa [8]. Nonetheless, according to the World Health Organization (WHO), only 1% of children below five years of age from lowest wealth quintiles make use of ITNs [1]. There are different reasons explaining these observations. First, in poorer households financial resources are scarce to invest in health care and prevention (e.g., treatment and prevention of malaria, including purchase of ITNs). Moreover, these households can usually not afford to improve housing quality (e.g., closing eves and screening of windows), which effectively protects against mosquitoes entering the house [9]. In general, the level of spending on prevention increases with socioeconomic status, although the relationships are not always direct and clear [10]. Second, knowledge on malaria transmission is essential for adequate use of preventive measures. The reduction of anophelines and culicines by ITNs plays an important role in community perceptions of ITN effectiveness [11]. Through an observable reduction of daily mosquito nuisance and clinical malaria episodes people perceive the direct benefits of bed nets and are more motivated to use them [12,13].

The national policy of malaria prevention in Côte d'Ivoire is based on use of ITNs, intermittent preventive treatment (IPT) with sulphadoxine pyrimethamine (SP) for pregnant mothers and management of the living environment. In 2008 an integrated vaccination campaign against measles was initiated in 84 health districts. This vaccination campaign also provided vitamin A and anti-helminthic drugs, together with free LLINs provided to children aged between nine months and five years. The national malaria control programme in Côte d'Ivoire, financed by the Global Fund to Fight AIDS, Tuberculosis and Malaria (Global Fund in short) has, as objective, to assure 80% coverage of the target population. Unfortunately, due to the political post-election crisis and security issues starting towards the end of November 2010 [14], Global Fund activities had to be suspended, including the large-scale distribution of LLINs to all households at risk.

The purpose of the current study was to assess knowledge and practice of malaria, with an emphasis on the use of LLINs in three rural communities of central Côte d'Ivoire, two of which had benefitted from free LLINs distributions by the national malaria control programme in previous years.

## Methods

### Study area and population

**Figure 1:**
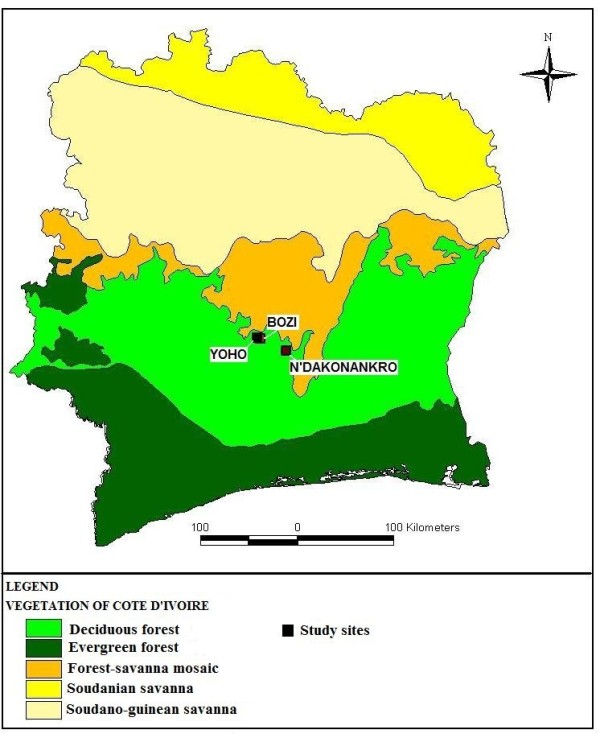
**Location of the three study villages in central Côte d'Ivoire**.

The study was carried out in central Côte d'Ivoire (zone of forests and southern part of the savannas) with a wet tropical type climate (Figure 1). There are four distinct seasons: (i) a short rainy season (March-May), (ii) a dry season lasting from May to July, (iii) a large rainy season (July-October), and (iv) another dry season from November to March.

Three villages were chosen for this study: Bozi, N'Dakonankro and Yoho, all located in central Côte d'Ivoire. Bozi is situated 27 km north-west from Yamoussoukro, the capital of the country. At the time of this study, there was a rural health centre, staffed with a nurse, a midwife and two medical assistants. According to a census made by the local health service, 1847 individuals were living in Bozi. There are two primary schools, the village has access to the power grid and households are served with running water. The village borders the Bandaman River with water used for irrigated rice farming in lowlands around the village. N'Dakonankro, inhabited by 827 individuals, is located south of Yamoussoukro. This village too is electrified, all households have running water and rice farming is an important agricultural activity. When the current survey took place, there was no school and no health centre; the nearest health centre is in Lobakro, some 3 km south of N'Dakonankro. Yoho is located 5 km from Bozi with a population of 1989 individuals. Yoho has one primary school, houses have yet to be electrified and there is no health centre, and hence health care is sought in Bozi or elsewhere. Water is provided by several manually operated pumps throughout the village.

In 2008, Bozi and Yoho received free LLINs from the national malaria control programme resulting in an increase of LLINs coverage from zero to 35.2% in Bozi and from zero to 10.2% in Yoho. N'Dankonankro did not benefit from this malaria control intervention.

### Cross-sectional questionnaire survey

In July 2009, the heads of randomly selected households were interviewed using a pre-tested questionnaire. Males usually head a household; in case they were absent, the wife of the head of household or another adult household member was interviewed instead. The questionnaire was administered by trained field enumerators at the home of the respondent. Overall, the questionnaire included 50 questions. The first section of the questionnaire pertained to demographic aspects of the household (e.g., number of household members, age and sex). The second section focussed on socioeconomic indicators, including possession of various household assets (e.g., radio, television, refrigerator, etc.) and household characteristics (e.g., wall and roof building materials). The third section investigated common knowledge and health seeking behaviour in relation to malaria and the use of bed nets and LLINs. With regard to malaria, information on perceived causes of malaria in young children, typical treatment behaviour of a sick person, preventive methods to avoid mosquito bites, including perceived advantages and disadvantages of LLINs, were investigated.

### Statistical analysis

Data were double entered and validated in EpiInfo version 6.04 (Centers for Disease Control and Prevention, Atlanta, USA). Statistical analyses were performed in STATA version 9 (Stata Corporation; College Station, USA). The socioeconomic position (SEP) of household was derived by principal component analysis (PCA), using selected housing characteristics and household assets ownership according to a previously used method [15], which was successfully adapted to local contexts of Côte d'Ivoire [16-19]. Household assets were transformed into dichotomous variables. Missing values were replaced by the mean of the respective asset [4]. The first principal component (PC) explained 31.5% of the overall variability. The asset scores were summed to a total score for each household. The assets with a positive score were associated with higher SEP, and a negative one was associated with lower SEP. The households were divided into wealth quintiles according to their total scores, so that each household was categorized as poorest, very poor, poor, less poor and least poor. Concentration index (CI) with respective standard error (SE) was calculated for people's knowledge of malaria, and ITN use in relation to their SEP [20]. The CI allows quantifying the degree of inequality in a particular variable in relation to SEP and the strength of the relationship, hence the direction of an association between the two variables [21]. A negative CI is in favour of poorer households. Hence, when the CI for a given variable differs from zero, this variable needs redistribution from the poorer half to the richer half, so that the effect on inequality can be reduced [22]. The *t*-test was used to show statistically significant associations between a variable and SEP. To compare groups, χ^2^, Fisher's exact test and Kruskal-Wallis test were used, as appropriate.

### Ethical considerations

This study was reviewed by the institutional scientific board of the Centre Suisse de Recherches Scientifiques en Côte d'Ivoire (CSRS; Abidjan, Côte d'Ivoire). After the purpose and procedures of the study were explained to the heads of the selected households or a household representative, written informed consent was obtained prior to asking any questions.

## Results

### Social and demographic characteristics of sampled households

There were 5815 individuals living in the 957 households sampled (Yoho: 2634 individuals; Bozi: 2354 individuals; N'Dakonankro: 827 individuals). As expected, a higher percentage of males (57.1%) than females (42.9%) was interviewed (Table 1), with a significant difference between villages (χ^2 ^= 23.30, degree of freedom (df) = 2, p < 0.001). In total, there were 283 (29.5%) households with children aged below one year and 566 (59.1%) households with children aged more than five years with statistical differences between villages (children aged < 1 year: χ^2 ^= 31.45, df = 2, p < 0.001; children aged above five years: χ^2 ^= 13.39, df = 2, p = 0.001). With regard to children aged between one and five years (520 households or 54.3%), no significant difference between villages was observed (χ^2 ^= 1.35, df = 2, p = 0.590). Matrimonial status was significantly different between villages (unmarried: χ^2 ^= 24.08, df = 2, p < 0.001; monogamous: χ^2 ^= 27.30, df = 2, p < 0.001; polygamous: χ^2 ^= 10.34, df = 2, p < 0.001; married and divorced: Fisher's exact test, p < 0.001). Significant village differences were also found for religion (Christian: χ^2 ^= 37.84, df = 2, p < 0.001; Muslim: Fisher's exact test, p < 0.001; animist: χ^2 ^= 44.73, df = 2, p < 0.001). The majority of heads of household obtained at least primary school education, whereas a large number of mothers with children aged below five years were illiterate (Yoho: 54.0%; Bozi: 49.8%; N'Dakonankro: 43.0%) and differences between villages were observed (household heads: Fisher's exact test, p < 0.001; mothers with children below the age of five years: Fisher's exact test, p = 0.001). Two thirds of the household heads were farmers, whereas the others earned their living as traders, teachers or artisans.

**Table 1:** Demographic characteristics of 957 selected households from the study villages Bozi, Yoho and N'Dakonankro, central Côte d'Ivoire

Variables	Bozi (%)	Yoho (%)	N'Dakonankro (%)	Total (%)
Sex of interviewee				
Male	226 (67.5)	259 (50.8)	61 (54.5)	546 (57.1)
Female	109 (32.5)	251 (49.2)	51 (45.5)	411 (42.9)
Age of children in the household				
> 5 years	211 (63.0)	276 (54.1)	79 (70.5)	566 (59.1)
1-5 years	214 (63.8)	234 (45.8)	72 (64.3)	520 (54.3)
< 1 year	93 (27.7)	159 (31.2)	31 (27.7)	283 (29.6)
Origin of head of household				
Native of the village	80 (24.7)	248 (49.9)	71 (68.3)	399 (43.1)
Non-native of the village	184 (56.8)	133 (26.7)	30 (28.8)	347 (37.5)
Foreign	60 (18.5)	116 (23.3)	3 (2.9)	179 (19.3)
Matrimonial status				
Unmarried	132 (40.6)	86 (17.1)	27 (24.1)	245 (26.0)
Monogamous married	163 (50.1)	340 (67.5)	68 (60.7)	571 (60.7)
Polygamous married	19 (5.8)	49 (9.7)	2 (60.7)	70 (7.4)
Divorced	1 (0.3)	14 (2.8)	9 (8.0)	24 (2.5)
Widower	10 (3.1)	15 (2.1)	6 (5.4)	31 (3.3)
Religion				
Christian	176 (54.1)	161 (32.9)	50 (45.4)	387 (41.8)
Muslim	71 (21.8)	117 (23.9)	4 (3.6)	192 (20.8)
Animist	62 (19.1)	181 (36.9)	54 (49.1)	297 (32.1)
Atheist	15 (4.6)	20 (4.1)	2 (1.8)	37 (4.0)
Fetich	0 (0)	11 (2.2)	0 (0)	11 (1.2)
Other	1 (0.3)	0 (0)	0 (0)	1 (0.1)
Education of head of household				
Illiterate	92 (28.5)	169 (33.3)	34 (30.9)	295 (31.4)
Primary	152 (47.1)	266 (52.5)	41 (37.3)	459 (48.8)
Secondary	68 (21.0)	68 (13.4)	31 (28.2)	167 (17.7)
University	11 (3.4)	4 (0.8)	4 (3.6)	19 (2.0)
Education of mother				
Illiterate	129 (49.8)	107 (54.0)	34 (43.0)	270 (50.4)
Primary	102 (39.4)	86 (43.4)	31 (39.2)	219 (40.9)
Secondary	27 (10.4)	5 (2.5)	14 (17.7)	46 (8.6)
University	1 (0.4)	0 (0)	0 (0)	1 (0.2)
Occupation				
Farmer	221 (64.2)	309 (70.1)	75 (61.1)	605 (66.8)
Stockbreeder/fisherman	6 (1.7)	17 (3.8)	2 (1.6)	25 (2.8)
Trader	52 (15.1)	54 (12.2)	23 (19.0)	129 (14.2)
Others (pensioner, teacher, artisan)	65 (18.9)	61 (13.8)	21 (17.4)	147 (16.2)

### Socioeconomic position (SEP)

Table 2 displays asset ownership and house characteristics of the 957 households, stratified by wealth quintiles. The highest unweighed scores were obtained for houses built with cement (0.34) and roof with metal sheet (0.34), followed by access to the power grid (0.30). The lowest scores were obtained for living in a house with bamboo walls (-0.33), followed by straw (-0.30) and plastic roof (-0.10).

**Table 2:** Asset list of 957 selected households stratified by socioeconomic position from the study villages Bozi, Yoho and N'Dakonankro, central Côte d'Ivoire

Asset	Total (%)	Unweighed score	Wealth quintiles				
			
			Poorest (n = 192)	Very poor (n = 201)	Poor (n = 182)	Less poor (n = 191)	Least poor (n = 191)
Electricity	489 (51.4)	0.3000	1.6	31.3	31.3	93.2	98.4
Water supply	298 (31.2)	0.2588	0.0	4.0	28.6	52.9	71.7
Radio	617 (65.4)	0.1394	42.1	58.1	59.0	75.5	92.1
Television	388 (40.7)	0.2836	0.5	23.6	19.3	63.9	95.8
CD reader	121 (12.9)	0.1932	0.0	4.6	4.4	10.0	46.7
DVD reader	188 (19.9)	0.2482	0.0	7.5	6.2	15.3	72.3
Refrigerator	64 (6.7)	0.1671	0.5	1.5	0.5	3.7	27.8
Ventilator	201 (21.4)	0.2854	0.0	2.0	4.6	16.1	85.0
Telephone	613 (64.9)	0.1796	40.5	49.7	59.5	77.9	96.8
Motorcycle	128 (13.5)	0.1283	5.2	5.0	10.7	11.6	35.3
Cooking energy							
Gas	19 (2.0)	0.1046	0.0	0.0	0.5	1.0	8.4
Coal	97 (10.1)	0.1510	0.0	6.0	6.0	8.4	30.4
House wall							
Cement	588 (61.4)	0.3435	0.0	28.4	86.3	96.9	98.9
Bamboo	354 (37.0)	-0.3397	99.5	66.2	12.1	3.1	1.0
House roof							
Metal sheet	624 (65.2)	0.3407	0.0	40.3	91.8	96.9	100
Straw	272 (28.4)	-0.3032	90.6	41.3	6.0	2.1	0.0
Plastic	61 (6.4)	-0.1046	9.4	18.4	2.2	1.0	0.0

### Bed net coverage

Most households in N'Dakonankro bought bed nets (41.0%), whereas in Bozi and Yoho fewer households reported having purchased bed nets (Bozi: 30.7%; Yoho: 21.1%) and the difference between villages was significant (χ^2 ^= 22.47, df = 2, p < 0.001). The percentage of households that owned at least one LLIN was 35.2% in Bozi and 10.7% in Yoho, after a net distribution campaign had been undertaken by the national malaria control programme in 2008. N'Dakonankro did not benefit from this free net distribution and, indeed, a lower coverage was found (7.1%). Among net owners, the rate of net usage was 47.8% in Bozi, 43.4% in Yoho, but only 8.8% in N'Dakonankro. These setting-specific percentages showed a highly statistically significant difference (χ^2 ^= 90.70, df = 2, p < 0.001). In Bozi 59.7% of the net owners reported having LLINs that were between six months and one year old, whereas in N'Dakonankro one quarter (25.8%) of the nets still in use had an age of at least two years. The wealthiest were more likely to use LLINs aged six months to one year and more than two years.

**Figure 2:**
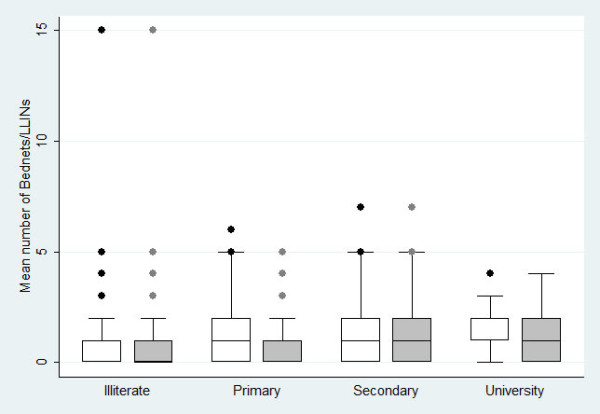
**Boxplot displaying bed nets (white coloured box) and LLINs (gray coloured box) number among 957 households, stratified by education levels of the head of households in central Côte d'Ivoire between September 2008 and September 2009**.

**Figure 3:**
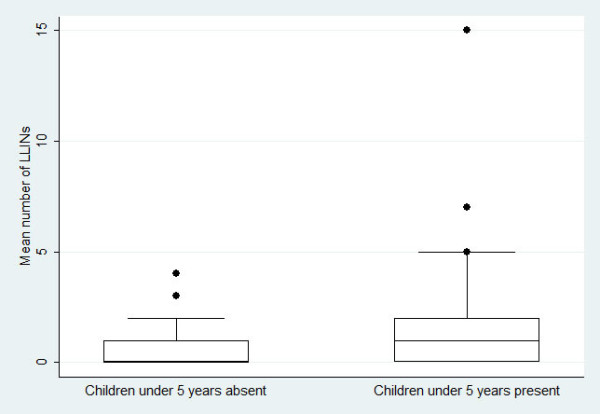
**Boxplot displaying the mean number of LLINs among 957 households, stratified by children under five years of age in central Côte d'Ivoire**.

As shown in Figure 2 the mean number of bed nets and LLINs within a household was related to the reported education attainment of the household head (bed nets: Kruskal-Wallis H = 12.69, df = 3, p = 0.005 and LLINs: H = 30.21, df = 3, p < 0.001). Additionally, the mean number of LLINs in the household was influenced by the presence of children aged below five years (Kruskal-Wallis H = 30.04, df = 1, p < 0.05) (Figure 3).

### Malaria knowledge and practice

Most respondents knew about malaria (Table 3), and this was significantly and positively associated to SEP. The main malaria symptoms identified by respondents were dark yellow urine, followed by fever or hot body, yellow eyes and vomiting, whereas only about 1% of households mentioned convulsion. Yellow eyes and dark yellow urine are signs of jaundice, which is common in cases of severe *Plasmodium falciparum *malaria [23]. A significant and positive association between fever or hot body, anaemia, yellow eyes and convulsion with SEP was found. Approximately 80% of N'Dakonankro households identified fever, yellow eyes and dark yellow urine as symptoms of malaria, whereas approximately 60% of households in Bozi and 30% in Yoho gave similar responses. Respondents reported mosquitoes, the sun and fever as main cause of malaria in children; however, a positive association with SEP was only found for mosquitoes, tiredness and dirty water.

**Table 3:** Malaria knowledge among 957 households according to their socioeconomic position (SEP) in the study villages Bozi, Yoho and N'Dakonankro, central Côte d'Ivoire

Variables	Total (%)	Wealth quintiles					**CI**^**a**^	**SE**^**b**^ (CI)	*t*-test (CI)
					
		Poorest (n = 192)	Very poor (n = 201)	Poor (n = 182)	Less poor (n = 191)	Least poor (n = 191)			
Malaria knowledge	90.8	84.9	87.6	94.5	91.1	96.3	0.023	0.007	3.30*
Malaria symptoms									
Yellow urine	57.0	59.4	52.2	57.1	55.5	61.3	0.009	0.016	0.58
Fever or hot body	55.6	35.4	43.8	59.3	66.0	74.3	0.144	0.034	4.28*
Yellow eyes	39.9	24.5	27.9	54.4	47.1	47.1	0.129	0.057	2.26*
Vomiting	17.4	16.7	18.4	17.0	14.1	20.9	0.019	0.037	0.53
Diarrhoea	1.8	2.1	1.5	1.1	1.6	2.6	0.051	0.092	0.55
Anaemia	1.7	0.5	0.0	1.1	2.1	4.7	0.496	0.093	5.32*
Convulsion	1.2	0.5	0.5	0.5	1.0	3.7	0.435	0.052	8.32*
Cough	1.0	0.0	0.5	1.6	2.1	1.0	0.281	0.205	1.37
Cause of malaria in children									
Mosquitoes	69.9	65.1	59.7	70.3	73.8	81.1	0.052	0.012	4.20*
Sun	34.5	40.6	29.3	34.1	32.5	36.1	-0.014	0.034	-0.42
Fever	17.8	15.6	18.4	19.2	14.7	20.9	0.031	0.033	0.93
Fatigue	5.6	4.2	1.5	3.3	6.3	13.1	0.317	0.085	3.75*
Bad food	4.1	1.0	2.5	6.6	5.8	4.7	0.207	0.135	1.54
Dirty water	1.7	1.0	1.5	1.1	1.0	3.7	0.230	0.108	2.13*
Sorcerer	0.1	0.0	0.0	0.0	0.5	0.0	0.403	0.357	1.13
First line-treatment									
Traditional medicines	47.9	59.9	53.7	47.2	44.0	34.0	-0.103	0.028	-3.73*
Modern medicines	26.1	10.4	22.4	26.9	29.3	41.9	0.214	0.075	2.88*
Traditional and modern medicines	15.0	11.5	11.9	16.5	18.8	16.7	0.093	0.027	3.48*
After first line-treatment									
Hospital	67.1	42.7	55.7	78.0	73.3	86.9	0.126	0.041	3.06*
Nothing	13.1	24.5	15.9	7.1	7.3	9.9	-0.233	0.056	-4.20*
Traditional healer	12.1	19.3	18.4	10.4	10.5	1.6	-0.287	0.122	-2.36*
Measures to avoid malaria									
Mosquitoes avoidance	60.8	60.4	53.2	56.6	62.3	71.7	0.041	0.023	1.76
Sun avoidance	27.1	35.4	24.9	25.8	19.9	29.3	-0.052	0.054	-0.95
Modern medicine	21.0	12.0	16.9	25.8	23.0	27.7	0.143	0.053	2.68*
Traditional cure	15.1	11.5	11.4	18.1	20.9	14.1	0.078	0.054	1.44
Herbal tea	8.1	6.8	9.4	11.0	5.2	8.4	-0.009	0.055	-0.16
Don't know	1.9	0.5	1.5	2.2	3.1	2.1	0.205	0.117	1.75
Child must avoid his totem	0.8	0.52	1.99	0.55	0.52	0.52	-0.140	0.135	-1.04

*Significant difference

^a^CI: concentration index

^b^SE: standard error

Most households reported to use traditional medicine as first-line treatment for malaria and less than one fifth of the households cited both traditional and modern drugs. When comparing between villages, it was found that in N'Dakonankro, households were more likely to use a combination of drugs locally purchased on the streets and modern drugs (χ^2 ^= 16.55, df = 2, p < 0.001) than traditional medicine (χ^2 ^= 8.83, df = 2, p = 0.012) as first-line treatment compared to Bozi and Yoho households. However, households in Yoho used less the combination of traditional and modern medicine (χ^2 ^= 42.08, df = 2, p < 0.001). The use of any form of traditional medicine was more often cited by households from N'Dakonankro than Bozi and Yoho (χ^2 ^= 52.15, df = 2, p < 0.001). Furthermore, 72.1% reported to visit hospital in case the employed first-line treatment was not efficacious. At the village level, in Bozi and N'Dakonankro, a large proportion of households reported to visit the hospital (χ^2 ^= 195.09, df = 2, p < 0.001) after failure of first-line treatments, while in Yoho, households preferred to consult traditional healers (Fisher's exact test, p < 0.001) or not taking any action (χ^2 ^= 39.01, df = 2, p < 0.001).

Treatment practice was significantly associated with SEP; households from the wealthiest group mentioned more often the use of modern (pharmaceutical or on drugs purchased from local street markets) medicine as first-line home treatment, whereas the poorest mentioned the use of any form of traditional medicine. If the first-line treatment failed, the wealthier groups preferred to visit the hospital, whereas the less wealthy groups preferred to visit a traditional healer or not to take any action.

More than half of the households cited mosquito avoidance as preventive measure against malaria. However, households from the least poor group thought that the use of medicine can avoid malaria, while the better-off groups believed that traditional medicine can help to avoid malaria. At the village level most households in Bozi and N'Dakonankro said that mosquitoes were responsible for malaria transmission compared to Yoho (χ^2 ^= 67.27, df = 2, p < 0.001) and among the preventive measures against malaria, 50% of N'Dakonankro households cited modern medicines (χ^2 ^= 126.03, df = 2, p < 0.001) and avoiding sun exposure (χ^2 ^= 42.13; df = 2, p < 0.001). No significant differences were observed between villages concerning the protection against mosquitoes (χ^2 ^= 1.80, df = 2, p = 0.392).

### LLINs knowledge and use

As shown in Table 4 LLINs knowledge was high. Information about LLINs was mainly given in hospital and broadcasted by television and radio. The poorest were more likely to report having received the information from friends, but the least poor mentioned having received the information through media, i.e., television and radio. At the village level, half of the heads of households in Bozi and N'Dakonankro reported to have been informed on LLINs primarily through hospitals, whereas in Yoho only 30% of the household heads reported hospitals as the main source of information. Television and neighbours played an important role in conveying malaria-related information in N'Dakonankro, compared to Bozi and Yoho.

**Table 4:** LLINs knowledge and use among 957 households, stratified by socioeconomic position (SEP) in the study villages Bozi, Yoho and N'Dakonankro, central Côte d'Ivoire

Variables	Total (%)	Wealth quintiles					**CI**^**a**^	SE^b ^(CI)	*t*-test (CI)
					
		Poorest (n = 192)	Very poor (n = 201)	Poor (n = 182)	Less poor (n = 191)	Least poor (n = 191)			
LLINs knowledge	87.4	81.2	87.6	86.3	84.3	97.4	0.027	0.015	1.75
Information process									
Hospital	41.0	40.6	33.3	45.0	37.7	48.7	0.039	0.028	1.41
Television	26.6	12.5	17.9	20.9	27.2	55.5	0.285	0.060	4.76*
Radio	23.5	17.7	23.4	22.0	28.8	25.6	0.073	0.028	2.56*
Friends	9.3	12.0	13.4	7.1	5.8	7.8	-0.138	0.027	-5.12*
Neighbour	6.0	5.7	4.5	5.5	8.4	5.8	0.053	0.040	1.33
Actions against mosquitoes									
ITNs	45.7	33.8	40.3	45.0	46.6	62.8	0.112	0.032	3.48*
Insecticide spray	34.2	42.2	31.3	24.2	31.4	41.4	-0.005	0.065	-0.07
Fumigating coils	24.2	18.2	21.9	25.8	30.4	25.1	0.074	0.034	2.18*
Bed nets	14.1	12.0	11.9	13.7	13.6	19.4	0.093	0.034	2.69*
Smoke by burning leave	0.6	1.0	1.0	0.5	0.5	0.0	-0.328	0.151	-2.17*
LLINs advantages									
Protection against mosquitoes	68.4	68.2	70.1	61.0	67.5	74.9	0.012	0.017	0.73
Protection against malaria	33.5	22.9	25.4	40.1	29.8	50.3	0.140	0.049	2.83*
Protection against others insects	6.3	2.1	2.5	4.9	8.4	13.6	0.368	0.062	5.96*
Non utilization of LLINs									
Expensive	21.9	33.8	23.4	24.7	16.7	11.0	-0.191	0.059	-3.26*
Family size	4.2	2.1	6.5	4.4	5.8	2.1	-0.010	0.143	-0.07
Not effective	0.8	0.0	0.0	0.5	2.1	1.6	0.498	0.206	2.42*
It's for one sleeping room	0.8	0.0	1.0	0.5	1.0	1.6	0.310	0.166	1.86
Does no protect against malaria	0.7	1.0	1.0	0.0	1.0	0.5	-0.110	0.100	-1.10
Mosquitoes bite outside LLINs	0.7	0.5	0.5	0.5	1.0	1.0	0.175	0.004	40.21*
LLINs side effect									
Heat	17.1	9.1	11.4	17.0	23.6	24.6	0.198	0.039	5.03*
Suffocation	7.4	1.6	3.0	9.3	8.9	14.7	0.343	0.113	3.03*
Unpleasant smell	1.9	0.5	0.0	0.5	4.2	4.2	0.488	0.134	3.63*

*Significant difference

^a^CI: concentration index

^b^SE: standard error

Measures against mosquitoes were associated to SEP. For example, use of bed nets, and more specifically ITNs, were more often cited by the least poor, whereas chasing away mosquitoes through smoke by burning plants (e.g., plant leaves) was reported by the poorer groups. At the village level, heads of households in Bozi reported using ITNs (χ^2 ^= 113.36, df = 2, p < 0.001) for protection against mosquitoes, while in Yoho and N'Dakonankro households reported more often the use of insecticide sprays (χ^2 ^= 14.50, df = 2, p = 0.001) and fumigating coils (χ^2 ^= 91.02, df = 2, p = 0.001).

The advantages for the use of LLINs mentioned by households included protection against mosquitoes (68.4%) and malaria (33.1%). The better-off groups reported more often protection against malaria and other insects as an advantage of LLINs. One fifth of the households and significantly poorer households stated that LLINs were expensive; less than 1% of households thought that LLINs were not efficient. The most widely reported negative effect or inconvenience arising from LLINs use was heat, followed by suffocation and unpleasant smell. At the village level, most of the heads of household in Bozi reported protection against malaria as a key advantage of LLINs (χ^2 ^= 139.92, df = 2, p < 0.001), in contrast to Yoho and N'Dakonankro households, who cited protection against mosquitoes more frequently (χ^2 ^= 5.65, df = 2, p = 0.059). The high costs of LLINs was cited by most Yoho and N'Dakonankro households as reason for not using LLINs (χ^2 ^= 32.85, df = 2, p < 0.001). The suffocation was a negative effect mentioned by one fifth of N'akonankro households versus less than one tenth of the households in Bozi and Yoho (χ^2 ^= 66.68, df = 2, p < 0.001).

## Discussion

The current study, carried out in three villages of central Côte d'Ivoire, determined whether social and economic factors influence the knowledge and preventive measures against malaria, placing particular emphasis on the use of LLINs. In two of the villages, LLINs were freely distributed by the national malaria control programme before the current investigation, whereas the third village did not benefit from this free bed net distribution campaign. A clear relationship was found between SEP and reported malaria symptoms, treatment behaviour and measures taken against mosquitoes. It was found that the education attainment of the household heads and whether children are present in a household are associated with LLINs utilization.

The main malaria symptoms reported by the heads of households or adult substitutes were fever or hot body. However, compared to previous studies carried out in central Côte d'Ivoire [18] and south-western Ethiopia [24], the frequency of these specific symptoms was lower. Convulsion was rarely mentioned as a symptom for malaria in the current setting. This observation confirms results obtained by Esse and colleagues, who described convulsion as a childhood health problem rather than a malaria-related symptom [18]. In other parts of Africa, convulsion was also not associated with malaria. For example, in the United Republic of Tanzania [25] and in Zambia [26], people recognized convulsions as a disease entity within the broad concept of epilepsy caused by supernatural forces, thus, requiring traditional healers for case management. In Ethiopia some caregivers associated convulsion to childhood malaria, which may indicate recognition of some features of severe malaria [24]. A history of fever is a widely used symptom in health facilities and at home that serves as an indicator for clinical malaria, and hence for initiating malaria treatment [27].

Household heads reported more often to use traditional medicine as first-line treatment before seeking care at a dispensary or a hospital. An important explanation of this observation is the lack of cash (traditional facilities often only require in-kind payments) [28]. The current results confirm this practice; wealthier households were more likely to report using modern pharmaceutical remedies and seeking care at official health services, whereas poorer households tended to use a broad range of traditional remedies. In general, modern medicine is more often used if it is readily available at home or if there is sufficient cash to purchase it. When positive experiences have been made with a specific drug before, it is likely to be used again. This is a common observation: home treatment is frequent, reaching up to 94% in rural Ghana and people - irrespective of SEP - use drugs within their immediate environment first, before seeking help/care and purchasing treatments [29,30]. In this context, the close proximity of one of the study villages (i.e., N'Dakonankro) to the capital of Yamoussoukro might have influenced people's practice with regard to treatment explaining the increased use of modern drugs sold by street vendors compared to traditional plants used in Yoho. Nonetheless, the results of this study suggest that there is still a major problem, particularly among poor population segments with the knowledge and adoption of the key malaria control strategy of early diagnosis and effective treatment.

More than two thirds of the household heads (69.9%) mentioned mosquitoes as the main cause of malaria in children. Mosquitoes were more frequently mentioned by wealthier households, suggesting better access to health information and education. This is in accordance with other studies pursued in Africa where mosquitoes were identified as a cause of malaria by people with better formal education and from better-off households [18,31,32]. Furthermore, findings presented here show that the use of LLINs was influenced by SEP, educational attainment of the heads of household and children under five years concurrently living in a household. In a study from Kenya, ITN use by children under the age of five years was positively associated with the caregiver's knowledge of ITNs, marital status and occupation [33]. In the current study from central Côte d'Ivoire, the better-off reported to have received information mainly through television and radio and their better formal education facilitated the understanding of the information provided. Actions against mosquitoes differed between socioeconomic groups. To fight against mosquitoes, almost half of the households reported to use ITNs, of which two thirds for protection against mosquitoes and one third for protection against malaria. The least poor mentioned more often to use LLINs to protect themselves. Thus, knowledge about mosquitoes as cause of malaria correlated with bed net use. In a study from western Ethiopia, the possession of bed nets, the willingness to pay for the nets and their actual use was associated with wealth status [34]. In another study from Ethiopia not owning ITNs was associated with unaffordability and no availability [35]. Conversely in Gabon, SEP was inversely related to bed net use [36]. This observation has been explained by the poorest being more bothered by insect nuisance than their richer counterparts, so they were more likely to use bed nets to protect themselves.

Two studied villages, namely Bozi and Yoho, received free LLINs from the national malaria control programme, whereas N'Dakonankro did not. Interestingly, N'Dakonankro and Yoho had approximately the same LLINs coverage, which was rather low (7.1% and 10.7%, respectively). The low and comparable rates of coverage can be explained by the large number of pregnant women of N'Dakonankro who visited hospitals in the city (Yamoussoukro) where they were given LLINs free of charge, while no such action was in place in the other study villages. Nonetheless, the utilization rate was higher (almost 50%) in the villages Bozi and Yoho, where the intervention by the national malaria control programme took place, compared to only 8.8% in N'Dakonankro. Furthermore, the mean number and use of LLINs in the current study was influenced by the education level of the head of households. This finding confirms results from Dike and colleagues [31], who observed that higher educational attainment was associated with a higher likelihood of households purchasing both treated and untreated bed nets, although in a study from western Côte d'Ivoire, Fürst and colleagues [19] found that the educational level of the respective heads of households had no influence. The current study further confirms that the mean number of LLINs in the household is governed by the presence of children younger than five years of age [37]. Results presented here further suggest that preventive measures against malaria (i.e., free distribution of LLINs) targeting particularly vulnerable groups, facilitated by the national malaria control programme might also have increased the malaria knowledge, and hence the use of LLINs.

## Conclusions

The findings of the current study carried out in rural parts of central Côte d'Ivoire confirm results from other settings [31], by reiterating that education and SEP play important roles in the control of malaria and the promotion of health in general. In particular, the findings of this study suggest that there is a need that current form of single or intermittent health education campaigns needs to be replaced with continuous learning processes that transmit and reinforce the knowledge of malaria transmission, the importance of prompt diagnosis and effective treatment and its prevention in order to increase equitable access to quality health care within a systemic approach to develop and strengthen health systems.

## List of abbreviations

CI: concentration index; DVD: digital versatile disc; ITN: insecticide-treated net; LLIN: long-lasting insecticidal net; SE: standard error; SEP: socioeconomic position

## Competing interests

The authors declare that they have no competing interests.

## Authors' contributions

AFO implemented the study, analysed the data and drafted the manuscript. GR contributed to the design of the study, analysis and interpretation of the data and the revision of the manuscript. CVAE contributed to the study implementation. JU contributed to the design of the study and assisted in the drafting and revision of the manuscript. MT and MD contributed to the design of the study and the revisions of the manuscript. BGK designed the study and assisted in all steps of study implementation, data analysis and interpretation and revision of the manuscript. All authors read and approved the final manuscript. AFO and BGK are guarantors of the manuscript
